# Biomass and Spore Yield of *Cercospora* aff. *canescens* by Submerged Fermentation: Defining and Optimizing Culture Conditions

**DOI:** 10.1007/s00284-026-05092-w

**Published:** 2026-08-26

**Authors:** Kátia de Lima Nechet, Alfredo José Barreto Luiz, Ana Júlia Dias dos Reis Barbosa, Gabriela Maria de Lima Dias, Bernardo de Almeida Halfeld-Vieira

**Affiliations:** Embrapa Meio Ambiente, Jaguariúna, São Paulo, Brazil

## Abstract

Morningglory species (*Ipomoea* spp.) are important weeds in Brazilian agriculture, particularly in non-burning sugarcane farming system. The fungus *Cercospora* aff. *canescens* has been selected as potential mycoherbicide; however, inoculum production is considered a challenge. The purpose of this study was to define and optimize culture conditions for biomass and spore yield of *Cercospora* aff. *canescens* CMAA 1444 in submerged fermentation. Initially, a Plackett–Burman experimental design with 12 treatments was conducted using a modified Czapek-Dox medium to screen the effect of these factors: carbon (dextrose): nitrogen (calcium nitrate) ratios (10:1, 50:1, and 100:1), pH (4, 7, and 8.5), light periods (0 = continuous dark, 12 h, and 24 h), magnesium sulphate (0.25 g.L^− 1^, 1 g.L^− 1^, and 2.5 g.L^− 1^) and potassium sulphate (0.25 g.L^− 1^, 1 gL^− 1^, and 2.5 g.L^− 1^) concentrations. Afterwards, individual assays were conducted in a completely randomized design using a broader range of C: N ratios (0:1; 20:1; 40:1; 60:1; 80:1; 100:1) and pH (3.5, 4, 4.5, 5, 5.5, 6, 6.5, 7, 7.5, 8; 8.5, and 9). Highest biomass and spore yield were obtained after 14 days of submerged fermentation. Optimal conditions shared by both responses included 24-h light period, magnesium sulphate at 0.25 g.L^− 1^, and potassium chloride at 0.25 g.L^− 1^. Maximum biomass (3.0 g) was achieved at C: N ratio 100:1 and pH = 8.5, whereas maximum spore yield (8.4 × 10^5^ spores.mL^− 1^) was achieved at C: N ratio 40:1 and pH = 5. These results represent an important step toward large-scale production of *Cercospora* aff. *canescens*, enabling its use as a mycoherbicide for the control of morningglories.

**Supplementary Information:**

The online version contains supplementary material available at https://doi.org/10.1007/s00284-026-05092-w.

## Introduction

The bioherbicide approach consists of the application of a plant pathogen, primarily fungi, through appropriate formulations to control weeds [1]. Bioherbicides have been considered an important tool for sustainable crop production within the One Health framework; however, despite their potential, only a limited number of products are currently registered [2]. One of the major constraints in their development is the mass production of biological control agents, particularly in fungal-based bioherbicides [3, 4]. Therefore, few commercial products formulated with fungal pathogens are available worldwide [5], and none specifically address Brazilian weed challenges [6], albeit bioinput adoption has been increasing annually, driven by both public policies through the National Bioinputs Program [7] and the increasing demand for expanded research initiatives in this field [8, 9].

In a previous study, *Cercospora* aff. *canescens* was selected as a potential mycoherbicide to control morningglory species (*Ipomoea* spp.). The pathogen induces irregular, pale brown spots on leaves of *I*. *nil*, *I*. *hederifolia*, and *I*. *grandifolia*, which subsequently turned yellow resulting in early abscission [10]. These species are considered hard-to-control weeds, particularly in non-burning sugarcane (*Saccharum officinarum*) production system, as they are able to germinate and overcome the straw layer deposited on the soil [11, 12]. Moreover, the presence of straw negatively affects chemical herbicide translocation, decreasing the efficacy of both pre- and post-emergent control strategies [13, 14]. Additionally, glyphosate-resistant biotypes of *I. nil* have already been reported, demonstrating the limited effectivity of one of the most widely adopted herbicide in Brazilian agriculture [15].

Given this scenario, morningglory was selected as a target weed for the development of a mycoherbicide based on propagules of *C.* aff. *canescens.* However, two major challenges have been identified: the requirement for a high inoculum density to induce plant defoliation and the difficulties in producing infective propagules from this isolate [16]. In general, the fungal genus *Cercospora* is notoriously difficult to sporulate in artificial media [17], which has historically limited their consideration as mycoherbicides candidates [18].

Thus, inoculum production represents one of the most significant bottlenecks in the early stages of mycoherbicide development. Currently, the production of infective inoculum of *C.* aff. *canescens* involves a two-step process: an initial submerged fermentation phase for biomass accumulation, followed by a solid-state fermentation phase for conidia production [16]. However, conidial yields remain low, and the need to integrate two distinct processes increases production time, infrastructure requirements, and labor intensity. In contrast, submerged fermentation provides faster growth kinetics, greater uniformity, better quality control, lower contamination risk, and enhanced scalability [19, 20].

Considering these advantages, the exclusive use of submerged fermentation may represent a more efficient strategy for inoculum production of *C.* aff. *canescens.* Therefore, the objective of this study was to define and optimize culture conditions for biomass and spore yield of *Cercospora* aff. *canescens* in submerged fermentation.

## Materials and Methods

### Origin of Isolate and Culture Conditions

The fungus *Cercospora* aff. *canescens* CMAA 1444 (GenBank accession number MG652650) was obtained from the Collection of Microorganisms of Agricultural and Environmental Importance (CMAA) at Embrapa Meio Ambiente in Jaguariúna, São Paulo State, Brazil. The isolate was grown on Potato Dextrose Agar (PDA) under 12-h photoperiod at 25 °C for seven days. In all assays described below, five disks (∅ = 15 mm) of the colony obtained were transferred to 250-ml Erlenmeyer flasks containing 100 ml of modified Czapek-Dox liquid medium to obtain seed culture.

### Definition of Culture Conditions for Biomass and Spore Yield

The following factors were used to select culture conditions for biomass and spore yield in submerged fermentation: Carbon: Nitrogen ratio (10:1, 50:1, and 100:1), pH (4, 7, and 8.5), light period (0 = continuous dark, 12 h, and 24 h), magnesium sulphate (0.25 g.L^− 1^, 1 g.L^− 1^, and 2.5 g.L^− 1^) and potassium sulphate (0.25 g.L^− 1^, 1 gL^− 1^, and 2.5 g.L^− 1^). The culture broth used was a modified Czapek-Dox medium composed of dextrose as carbon source (C_6_H_12_O_6_H_2_O), calcium nitrate as nitrogen source (Ca(NO_3_)_2_.4H_2_O − 1 g.L^− 1^), magnesium sulphate (MgSO_4_7H_2_O), potassium phosphate (K_2_HPO_4_), potassium chloride (KCl- 0.5 g.L^− 1^), and iron sulphate (FeSO_4_H_2_O- 0,364 g.L^− 1^). The quantities of dextrose, magnesium sulphate, and potassium phosphate varied according to the assay and are specified in Table 1. A Plackett–Burman (PB) experimental design was used and consisted of 12 treatments (PBD 12), plus three central points corresponding to the standard treatment (Tables 1 and 2).

**Table 1:** Values of factors and levels (low, central, and high) of the experimental Plackett–Burman Design for selection of culture conditions for biomass and spore yield of *Cercospora* aff. *canescens*

Factors	Code	Levels		
		-1(low)	0 (central)	1 (high)
C: N	X_1_	10:1 (4.5 g.L^− 1^)	50:1 (22.5 g.L^− 1^)	100:1 (45 g.L^− 1^)
pH	X_2_	4	7	8.5
Light period (h)	X_3_	0	12	24
Magnesium sulphate (g.L^− 1^)	X_4_	0.25	1	2.5
Potassium sulphate (g.L^− 1^)	X_5_	0.25	1	2.5

**Table 2:** Experimental design of 15 assays to select culture conditions: C:N (X_1_), pH(X_2_), light period (X_3_), magnesium sulphate (X_4_) and potassium chloride (X_5_) for biomass and spore yield of *Cercospora* aff. *canescens*

Assays	X_1_	X_2_	X_3_	X_4_	X_5_
**1**	1	-1	1	-1	-1
**2**	1	1	-1	1	-1
**3**	-1	1	1	-1	1
**4**	1	-1	1	1	-1
**5**	1	1	-1	1	1
**6**	1	1	1	-1	1
**7**	-1	1	1	1	-1
**8**	-1	-1	1	1	1
**9**	-1	-1	-1	1	1
**10**	1	-1	-1	-1	1
**11**	-1	1	-1	-1	-1
**12**	-1	-1	-1	-1	-1
**13**	0	0	0	0	0
**14**	0	0	0	0	0
**15**	0	0	0	0	0

-1 low level; 0 central level; 1 high level

In all assays, five mycelial disks from CMAA 1444 were transferred to Erlenmeyer flasks containing 100 mL of modified Czapek-Dox liquid medium, prepared according to combinations of culture conditions shown in Table 2. The flasks were kept in an orbital shaker at 150 rpm and at 25 °C. After 7, 14, and 21 days of fermentation, the biomass was separated from the liquid fraction by filtration using a Whatman N° 1 paper filter, and dried at 70 °C until reaching constant weight. The liquid fraction was then centrifuged at 5000 rpm for 5 min at 25° C to obtain a 5 mL of concentrated suspension. The inoculum concentration (spores.mL^− 1^) was quantified in a Neubauer chamber. Each assay consisted of three sampling periods (7,14, and 21 days), with five flasks per period, each representing one experimental unit.

The data was analyzed through multivariate analysis, using the stepwise regression analysis procedure of SAS 9.4 [22]. In the present case, causes of variation were included in the model only if they were significant at the 30% level (*p* < 0.3) and were retained only if they remained significant at the 10% level (*p* < 0.1). The analysis considered as causes of variations the individual factors, their square numbers, and all the simple interactions. Therefore, each cause of variation was explained by the model:

$$\begin{aligned}\rm Y&=\rm f (X_1; X_2; X_3; X_4;X_5; X_1^2; X_2^2; X_3^2; X_4^2;\\&\quad \rm X_5^2; X_1^*X_2; X_1^*X_3; X_1^*X_4; X_1^*X_5; X_2^*X_3;\\&\quad\rm X_2^*X_4; X_2^*X_5; X_3^*X_4; X_3^*X_5; X_4^*X_5)\end{aligned} $$

### Production of Seed Culture for the Individual Assays

The production of seed culture followed the methodology of Nechet et al. (2024) [21] with modifications. The isolate CMAA 1444 was grown on PDA under a 12-h photoperiod at 25 °C for seven days. After this period, ten mycelial disks were transferred to 500-mL Erlenmeyer flasks containing 300 mL of modified Czapek-Dox liquid medium using the culture conditions selected for biomass production on the previous assay described (C: *N* = 100:1; dextrose (45 g.L^− 1^), calcium nitrate (1 g.L^− 1^), pH 4, magnesium sulphate (1 g.L^− 1^), potassium sulphate (0,25 g.L^− 1^). The Erlenmeyer flasks were placed in an orbital shaker (150 rpm) at 25 °C, under continuous light, for seven days. The biomass was separated from the liquid fraction by filtration, and aliquots containing 5 mL of the filtrate, adjusted to 12,850 cfu.mL^− 1^, were used as seed culture.

### Effect of pH on the Spore Production of *Cercospora* aff. *canescens*

The assay was carried out in 250-mL Erlenmeyer flasks containing 45 mL of modified Czapek-Dox liquid medium and 5 mL of seed culture. The culture conditions were the ones previously selected for spore yield assay (C: *N* = 10:1, dextrose (4.5 g.L^− 1^), calcium nitrate (1 g.L^− 1^), magnesium sulphate (0.25 g.L^− 1^), potassium sulphate (0.25 g.L^− 1^)), varying only the pH values (3.5, 4, 4.5, 5, 5.5, 6, 6.5, 7, 7.5, 8; 8.5, and 9). The flasks were placed in an orbital shaker (150 rpm) at 25 °C, under continuous light. After 7 and 14 days of submerged fermentation, the flasks were submitted to ultrasound for 5 min and filtered to separate the biomass from the liquid fraction. The liquid fraction was centrifuged at 5000 rpm for 5 min at 25° C to obtain a 5-mL concentrated suspension. The inoculum concentration was quantified in a Neubauer chamber (spores m.L^− 1^).

The assay followed a completely randomized design with 12 treatments (pH values) and four replications. Each replication consisted of a 1-mL sample obtained from the concentrated suspension, evaluated in a Neubauer chamber. The same experiment was conducted twice on different dates.

A third assay was later performed considering only the following pH values: 3.5, 4, 4.5, 5, 5.5, and 6, following the same methodology and evaluation procedures.

Spore yield data (spores.mL^− 1^) were tested for normality using the Shapiro-Wilk test. Data deviating from a normal distribution were subjected to Box-Cox transformation. Both sets of data were analyzed by ANOVA using SAS 9.4 [22], and means submitted to Duncan´s test (*p* < 0.05).

### Effect of C: N Ratio on the Spore Production of *Cercospora* aff. *canescens*

The assay was conducted in 250-mL Erlenmeyer flasks containing 45 mL of modified Czapek-Dox liquid medium and 5 mL of seed culture. The medium was contained magnesium sulphate (0.25 g.L^− 1^), potassium sulphate (0.25 g.L^− 1^), calcium nitrate (1 g.L^− 1^), pH 5. The C: N ratio varied according to Table 3. Culture conditions and methodology of evaluation were identical to those previously described. Evaluation was performed on the 14th day of submerged fermentation.

The experiment followed a completely randomized design with six treatments (C: N ratios) and four replications, each represented by a Neubauer chamber counting (Table 3). The same experiment was conducted twice on different dates.

**Table 3:** Carbon concentrations (g.L^− 1^) of treatments with different C: N ratio

C: *N* ratio	Carbon Concentration (g.L^− 1^)
0:1	0
20:1	9.00
40:1	18.00
60:1	27.00
80:1	36.00
100:1	45.00

Spore yield data (spores m.L^− 1^) were tested for normality using the Shapiro-Wilk test. Both sets of data were analyzed by ANOVA using SAS 9.4 [22] and means submitted to Duncan´s test at 5% significance. Joint analysis of variance was performed according to Gomes (2009) [23] based on Error Mean Square ratio lower than 4:1 between the two assays.

## Results

### Definition of Culture Conditions for Biomass and Spore Yield

Different models were adjusted for both parameters evaluated - biomass and spore yield - and for each sampling period (7,14, and 21 days) (Supplementary Information).

The optimum factors for the maximum values of biomass (g) and spore yield (spores.mL^− 1^) in each sampling period (7, 14, and 21 days) are presented in Table 4.

**Table 4:** Optimum factors for maximum biomass (g) and spore yield (spores.mL^− 1^) after 7, 14, and 21 days of submerged fermentation of *Cercospora* aff. *canescens*

Days	Factors		Biomass (g)		Spore yield (spores.mL^− 1^)
7	C: N ratio	100	2.4	100	1.6 × 10^5^
	pH	4		4	
	light period	24		24	
	magnesium sulphate	1		0.25	
	potassium chloride	0.25		0.25	
14	C: N ratio	100	3.0	10	14 × 10^5^
	pH	8.5		4	
	light period	24		24	
	magnesium sulphate	0.25		0.25	
	potassium chloride	0.25		0.25	
21	C: N ratio	75	3.0	10	11 × 10^5^
	pH	4		8.5	
	light period	0		24	
	magnesium sulphate	2.5		0.25	
	potassium chloride	2.5		0.25	

The highest biomass and spore yield values, estimated by using the adjusted equations, were obtained after 14 days of submerged fermentation. For all data collected in this sampling period, the levels of light period (24 h), magnesium sulphate (0.25 g.L^− 1^), and potassium chloride (0.25 g.L^− 1^) were the same. Besides, these levels were also the most frequent combination that led to the maximum biomass and spore yield of *Cercospora* aff. *canescens* and were therefore fixed in the following adjusted equations:

$$\begin{aligned}&\rm Biomass\, (14 days) = 1.14276 + (0.33568^*0.25) + (0.00066326^*X_1^*24)\\&\quad\rm - (0.00361^*X_1^*0.25) + (0.00217^*X_2^*24) - (0.01021^*X_3^*0.25)\\&\qquad\rm - (0.01879^*24^*0.25) + (0.10823^*0.25^*0.25);\end{aligned} $$

$$\rm Biomass\, (14 days) = 1.05944 + (0.01502^*X_1) + (0.05208^*X_2)$$

**Fig. 1:**
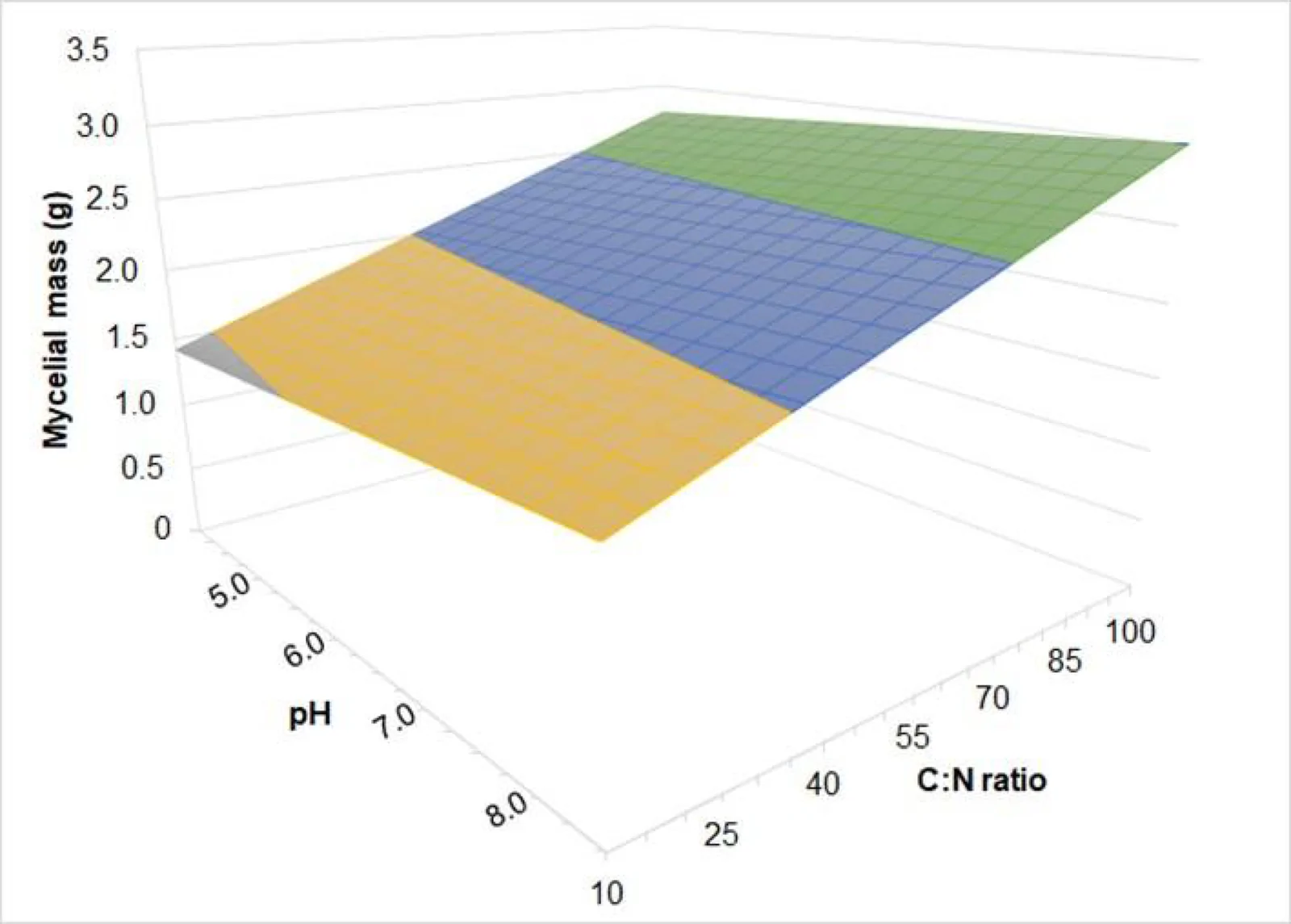
Response surface for the biomass (g) of *Cercospora* aff. *canescens* as a function of C: N ratio and pH after 14 days of submerged fermentation

This equation is represented in Fig. 1 as a linear surface without peaks or valleys, and the maximum biomass value was obtained using a C: N ratio = 100 and pH = 8.5.

$$\begin{aligned}&\rm Spore\, production (14\, days) = 3.84259 + (2.99519^*24) \\&\quad+ (0.19509^*242) - (0,02873^*X_1^*24) - (0.17690^*X_2^*24)\\&\qquad - (1.50292^*24^*0.25) - (1.27047^*24^*0.25)\end{aligned} $$

$$\rm Spore\, yield (14\, days)=171.45865 - (0.68952^*X_1) - (4.2456^*X_2)$$

**Fig. 2:**
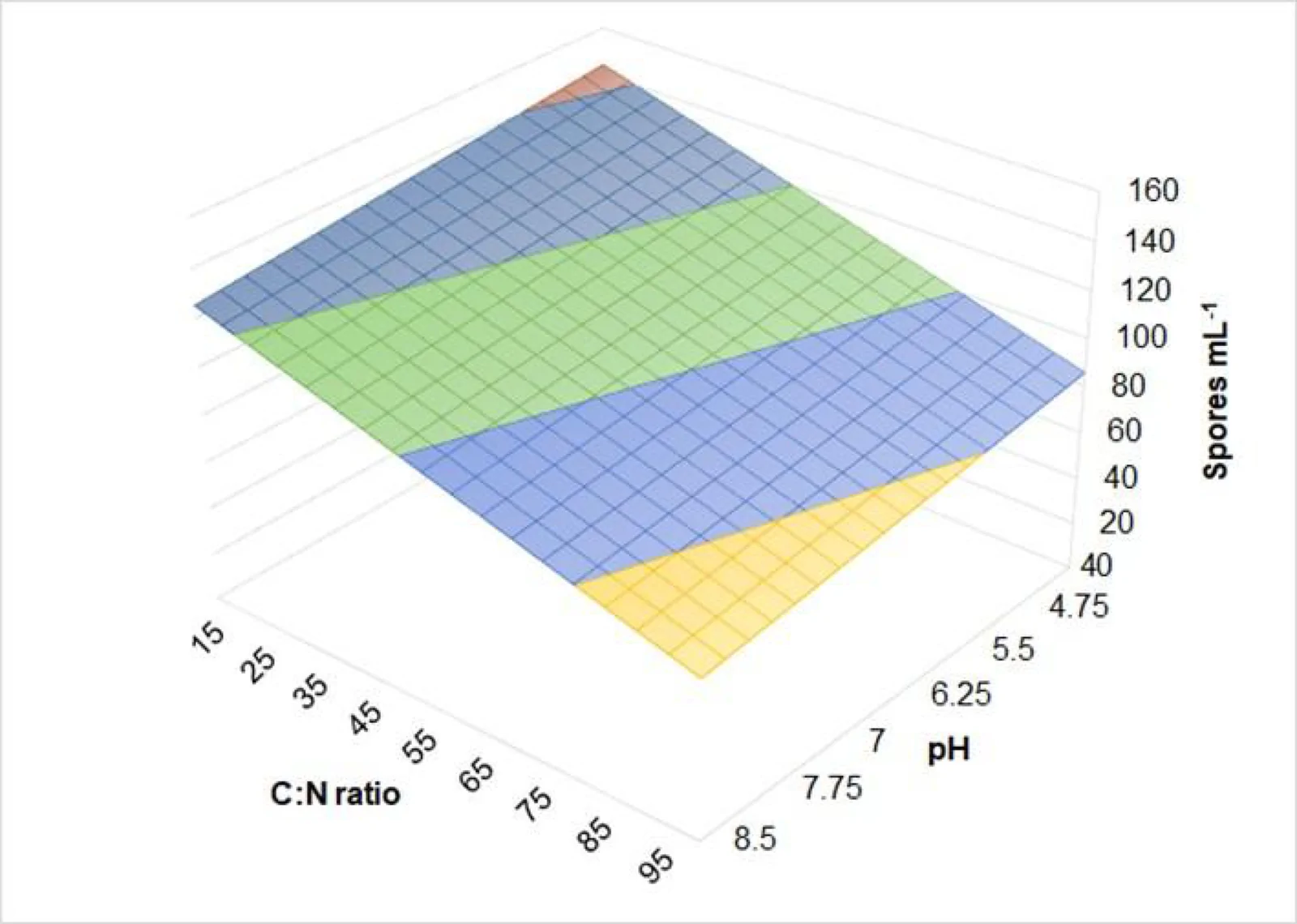
Response surface for the spore yield (spores.mL^− 1^) of *Cercospora* aff. *canescens* as a function of C: N ratio and pH after 14 days of submerged fermentation

This equation is represented in Fig. 2 as a linear surface without peaks or valleys, and the maximum spore yield value was obtained using a C: N ratio = 10 and pH = 4.

Based on these results, the combination of C: N ratio = 100, magnesium sulphate (0.25 g.L^− 1^), potassium chloride (0.25 g.L^− 1^), pH = 8, and light period (24 h) was selected for biomass production of seed culture. Considering that the most relevant outcome is the spore yield, C:N ratio and pH were used as variables in the following experiments conducted for 7 and 14 days of submerged fermentation.

### Effect of pH on the Spore Production of *Cercospora* aff. *canescens*

**Fig. 3:**
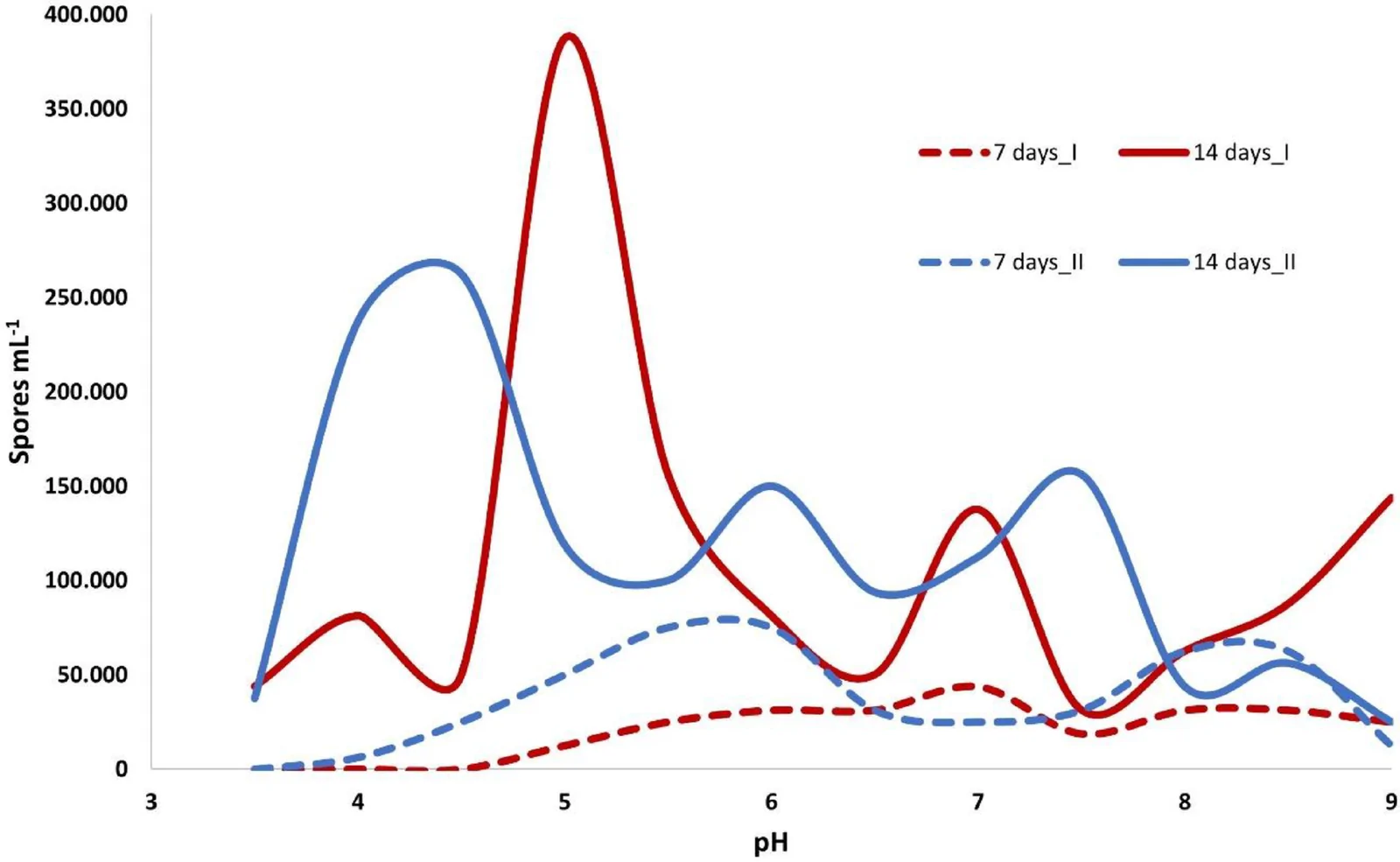
Spore yield (spores.mL^− 1^) after 7 and 14 days of submerged fermentation of *Cercospora* aff. *canescens* using modified Czapek-Dox medium with different levels of pH

Spore yield (spores.mL^− 1^) of *Cercospora* aff. *canescens* was observed after 7 and 14 days of submerged fermentation. In assay I, the maximum spore yield observed was 3.8 × 10^5^ spores.mL^− 1^ at pH 5 after 14 days, and 4.3 × 10^4^ spores.mL^− 1^ at pH 7 after 7 days. In assay II, the maximum spore yield observed was 2.6 × 10^5^ spores.mL^− 1^ at pH 4.5 after 14 days, and 7.5 × 10^4^ spores.mL^− 1^ at pH 5.5 and 6 after 7 days. In both assays, the maximum spore yield of *Cercospora* aff. *canescens* was observed after 14 days of submerged fermentation (Fig. 3).

**Fig. 4:**
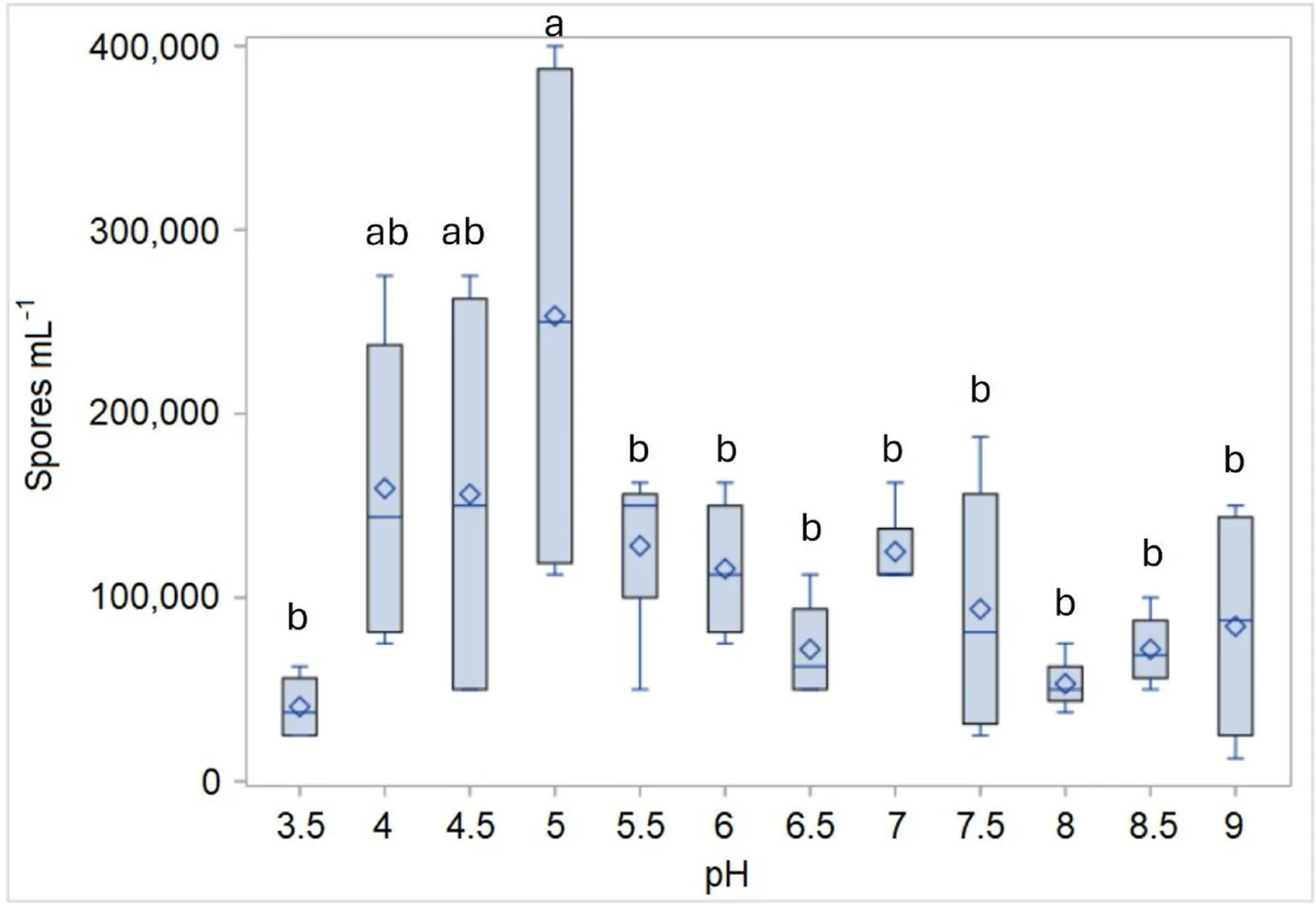
Spore yield distribution (spores.mL^− 1^) after 14 days of submerged fermentation of *Cercospora* aff. *canescens* using modified Czapek-Dox medium at different pH levels. Data represent the means of two assays. Means followed by the same letter do not differ significantly according to Duncan´s test (*p* < 0.05)

The distribution of spore yield for each pH level, based on the mean values from both assays, is presented in Fig. 4. A significant difference in spore yield was observed among pH levels. The maximum mean spore yield was 2.53 × 10^5^ spores.mL^− 1^ at pH 5, followed by 1.59 × 10^5^ spores.mL^− 1^ and 1.56 × 10^5^ spores.mL^− 1^ at pH 4 and 4.5, respectively. Considering that the means obtained at pH 4 and 4.5 were not different from the other pH levels tested, a third assay was carried out using pH 3.5, 4, 4.5, 5, 5.5, and 6 as treatments.

**Fig. 5:**
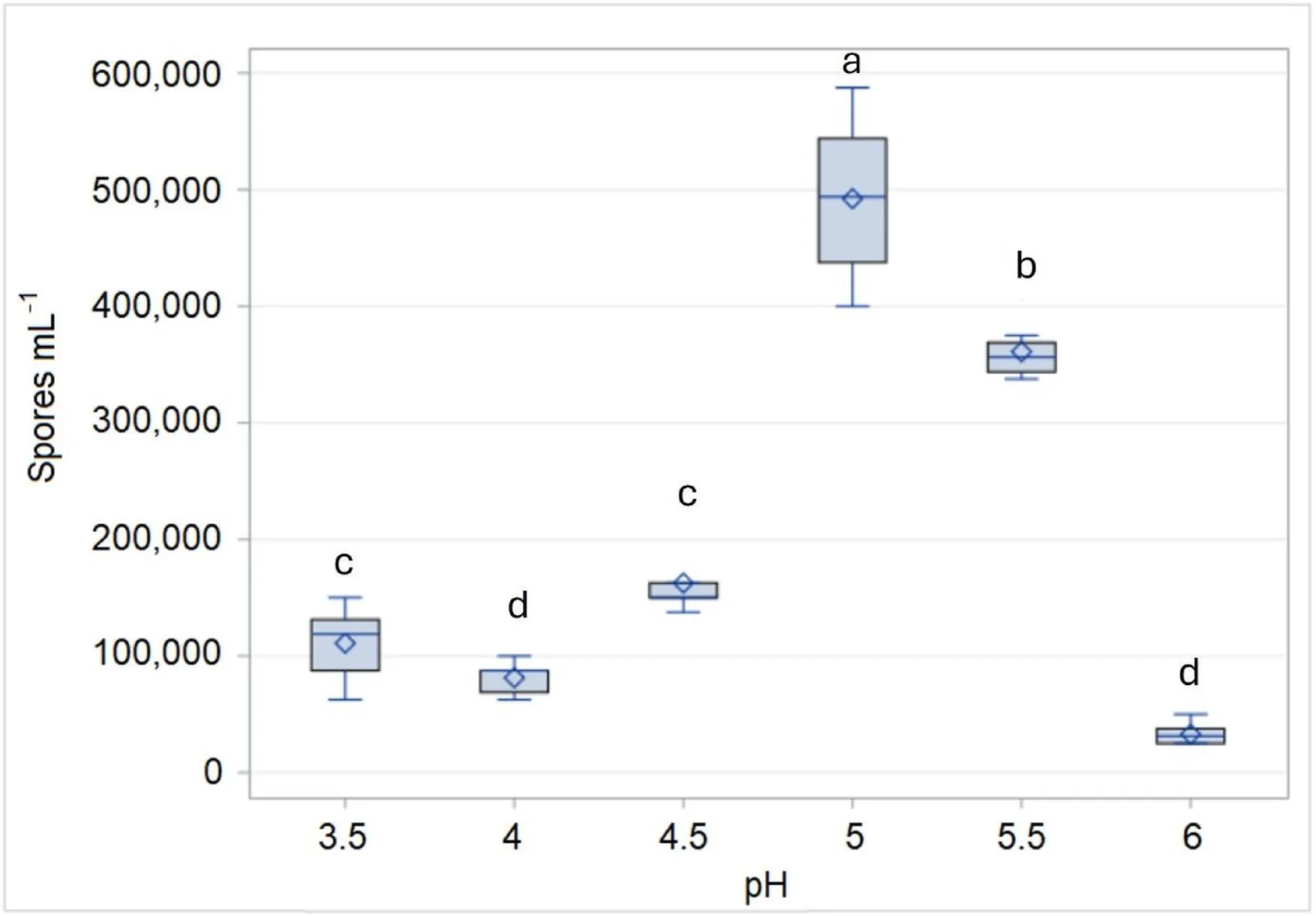
Spore distribution (spores.mL^− 1^) after 14 days of submerged fermentation of *Cercospora* aff. *canescens* using modified Czapek-Dox medium at pH 3.5, 4, 4.5, 5, 5.5, and 6. Means followed by the same letter do not differ significantly according to Duncan´s test (*p* < 0.05)

In the third assay, a significant difference in spore yield among the treatments was observed (Fig. 5). The mean spore yield was highest at pH 5 (4.9 × 10^5^ spores.mL^− 1^), followed by pH 5.5 (3.6 × 10^5^ spores.mL^− 1^). Spore yield at pH 3.5 and 4.5 were similar, ranging from 1.1 × 10^5^ to 1.6 × 10^5^ spores.mL^− 1^. The lowest spore yield was observed at pH 4 and pH 6 (3–8 × 10^4^ spores.mL^− 1^). Therefore, pH 5 was selected for the subsequent assays.

### Effect of C: N Ratio on the Spore Yield of *Cercospora* aff. *canescens*

The C: N ratio had a significant effect on spore yield of *Cercospora* aff. *canescens* with consistent differences observed across all tested ratios in both assays and in joint analysis (Table 5). The highest spore yield were observed at C: N ratio 40:1 in both assay I (10.4 × 10^5^ spores.mL^− 1^) and assay II (6.5 × 10^5^ spores.mL^− 1^). The second highest spore yield was 2.4 × 10^5^ spores.mL^− 1^ at C: N ratio 80:1 which was significantly different from the other C: N ratios. No spore yield occurred at 0:1 C: N ratio (Table 5).

**Table 5:** Spore yield (spores.mL^− 1^) of *Cercospora* aff. *canescens* after 14 days of submerged fermentation using modified Czapek-Dox medium with different C: N ratios. Means followed by the same letter in the column do not differ significantly according to Duncan´s test (*p* < 0.05)

C: *N* ratio	Assay I	Assay II	Mean
40:1	10.4 × 10^5^ a	6.5 × 10^5^ a	8.4 × 10^5^ a
80:1	3.5 × 10^5^ b	1.4 × 10^5^ b	2.4 × 10^5^ b
60:1	1.03 × 10^5^ c	1.5 × 10^5^ b	1.2 × 10^5^ c
20:1	1.2 × 10^5^ c	1.3 × 10^5^ b	1.2 × 10^5^ c
100:1	0.4 × 10^5^ d	0.53 × 10^5^ c	0.46 × 10^5^ cd
0:1	0 d	0 d	0 d

## Discussion

In the present study, the results indicate suitable culture conditions to optimize the biomass and spore yield of *Cercospora* aff. *canescens* through submerged fermentation. This study represents a novel contribution since the inoculum production of this biocontrol agent has been identified as a major obstacle to developing a mycoherbicide for controlling morningglory species.

The C: N ratio and initial pH were identified as the main parameters for optimizing both biomass growth and spore yield of *Cercospora* aff. *canescens* (Figs. 1 and 2; Equations provided in the Supplementary Information). The use of a modified Czapek-Dox medium with the highest C: N and pH levels tested resulted in maximum biomass production but did not promote maximum sporulation after 14 days (Table 4). This finding is consistent with numerous studies showing that the nutrient conditions required to optimize biomass growth differ from those that favor spore production [24–26].

The maximum sporulation of *Cercospora* aff. *canescens* (8.4 × 10^5^ spores.mL^− 1^) was achieved after 14 days using modified Czapek-Dox medium with initial pH 5 and C: N ratio 40:1 (Figs. 4 and 5; Table 5). Similar studies have reported the influence of pH and C: N ratio on the sporulation of fungi in submerged fermentation for the production of inoculum for biological control agents [27–29]. In general, these studies highlight that different microorganisms have specific individual requirements to optimize the production of infective structures, which is influenced by various factors [30], including the species-specificity characteristics observed among cercosporoid fungi [17].

The C: N ratio of 40:1 required for maximum spore yield of this fungus has also been reported for the nematophagous fungus *Pochonia chlamydosporia* and for the entomopathogenic fungus *Beauveria bassiana* [31]. Carbon and nitrogen sources are also important factors affecting sporulation. Carbon is one of the most important components for microbial growth, and most fungi can metabolize several monosaccharides, generally preferring glucose over other sugars [32, 33]. In the current study, glucose (dextrose) was used as carbon source, and calcium nitrate as nitrogen source; however, a wide range of organic compounds can be metabolized by fungi to stimulate sporulation [34, 35].

In our study, an initial pH of 5 promoted the highest spore yield of *Cercospora* aff. *canescens*. Similar studies on fungi also demonstrated that a pH range from 4 to 6 in submerged fermentation produced highest sporulation [36, 37]. During cultivation, pH may shift due to depletion of nutrients and accumulation of metabolites in the broth, affecting microbial growth [38]. Despite the pH drift from 5 to 7 observed in this study, no negative impact on spore yield was detected.

Submerged fermentation is a well-established process for the production of commercial biopesticides. However, in Brazil, most fungal propagules used as biological control agents are still produced on solid substrate. The ability to obtain high spore yields of *Cercospora* aff. *canescens* through submerged fermentation can overcome the major constraints for mass production of this fungus as mycoherbicide. Previously, a biphasic technique was employed for inoculum production of *C*. aff. *canescens* [16]; however, this method is time-consuming and may increase the risk of contamination during the second stage of the process. This study demonstrated that strategic adjustments in submerged fermentation parameters help overcome not only the reliance on a two-stage fermentation process but also the challenges of promoting sporulation of filamentous fungi in liquid media [39].

Selecting suitable factors during shake-flask stage is a critical step in the scale-up process. The present study defines specific conditions to enhance spore yield in *C*. aff. *canescens* isolate. Maximum spore production reached 8.4 × 10^5^ spores.mL^− 1^ under optimal conditions of a C: N ratio of 40:1, magnesium sulphate at 0.25 g.L^− 1^, potassium chloride at 0.25 g.L^− 1^, pH 5, and a continuous light exposure for 24 h. These findings pave the way for an effective submerged fermentation medium that supports large-scale inoculum production of *C*. aff. *canescens* as a mycoherbicide for the control of morningglories.

## Supplementary Information

Below is the link to the electronic supplementary material.

Supplementary Material 1

## Data Availability

The datasets generated during the current study are available from the corresponding author on reasonable request.
