# Supplementary material for: Biomass and Spore Yield of Cercospora aff. canescens by Submerged Fermentation: Defining and Optimizing Culture Conditions
Source: Curr Microbiol. 2026 Aug 26;83(10):533. doi: 10.1007/s00284-026-05092-w (PMC13518433; doi:10.1007/s00284-026-05092-w)
Supplement: Supplementary file 1 [file 284_2026_5092_MOESM1_ESM.pdf]

Supplementary Information

Equations considering 7 days of submerged fermentation of *Cercospora* aff. *canescen*s, the models adjusted for biomass ($p < 0.0001$; $R^2 = 0.8020$) and spore yield ($p < 0,0001$; $R^2 = 0.7282$):

Biomass (7 days) = $1.14334 + (0.00261*X_1) + (0.04299*X_3) - (0.16681*X_4) + (0.00256*X_1*X_4) - (0.01820*X_3*X_5) + (0.16590*X_4*X_5)$

Spore yield (7 days) = $0.63930 + (0.08899*X_1) + (0,02844*X_3^2) - (0.01884*X_1*X_2) + (0.28849*X_2*X_4) - (0.15756*X_2*X_5) - (0.34372*X_3*X_4) - (0.10556*X_3*X_5) - (1.37695*X_4*X_5)$

Equations considering 14 days of submerged fermentation of *Cercospora* aff. *canescen*s, the models adjusted for biomass ($p < 0.0001$; $R^2 = 0.6995$) and spore yield ($p < 0.0001$; $R^2 = 0.8593$):

Biomass (14 days) = $1.14276 + (0.33568*X_5) + (0.00066326*X_1*X_3) - (0.00361*X_1*X_5) + (0.00217*X_2*X_3) - (0.01021*X_3*X_4) - (0.01879*X_3*X_5) + (0.10823*X_4*X_5)$

Spore yield (14 days) = $3.84259 + (2.99519*X_3) + (0.19509*X_3^2) - (0.02873*X_1*X_3) - (0.17690*X_2*X_3) - (1.50292*X_3*X_4) - (1.27047*X_3*X_5)$

Equations considering 21 days of submerged fermentation of *Cercospora* aff. *canescen*s, the models adjusted for biomass ($p < 0.0001$; $R^2 = 0.4275$) and spore yield ($p < 0.0001$; $R^2 = 0.8798$) :

Biomass (21 days) = $0.48748 + (0.05657*X_1) - (0.00038863*X_1^2) + (0.0789*X_4*X_5)$

Spore yield (21 days) = $1.61306 + (0.22024*X_3^2) - (0.01480*X_1*X_3) + (0.36650*X_2*X_4) - (1.36363*X_3*X_4) - (0.79703*X_3*X_5)$

where:

X_1 = C:N

X_2 = pH

X_3 = light period

X_4 = magnesium sulphate

X_5 = potassium chloride
